# Short-Segment Coil Embolization Using a Double-Balloon Technique in an Experimental Vascular Model

**DOI:** 10.1007/s00270-017-1589-1

**Published:** 2017-02-08

**Authors:** Daisuke Yunaiyama, Toru Saguchi, Tomohisa Moriya, Natsuhiko Shirota, Jun Otaka, Koichi Tokuuye, Yuichi Nagakawa, Akihiko Tsuchida, Kazuhiro Saito

**Affiliations:** 1 Department of Radiology, Tokyo Medical University, 6-7-1 Nishishinjuku, Shinjuku-ku, Tokyo, Japan; 2 Department of Gastrointestinal and Pediatric Surgery, Tokyo Medical University, Tokyo, Japan

**Keywords:** Coil embolization, Balloon catheter, Experiment, Double-balloon technique

## Abstract

**Purpose:**

To evaluate the feasibility of short-segment coil embolization between 2 balloons for tight packing in an experimental vascular model.

**Materials and Methods:**

Three coil embolization techniques were performed by 5 interventional radiologists as follows: (1) proximal balloon technique (proximal BT) which involved proximal balloon inflation and coil deployment over the balloon, (2) distal balloon technique (distal BT) which involved distal balloon inflation and coil deployment at the proximal side of the inflated balloon, and (3) double-balloon technique (DBT) which involved coil deployment between 2 inflated balloons. We used a 10-mm-diameter and 200-mm-long hydrocoil. The distance between the 2 inflated balloons was set at 5 mm in the perfused tube, and each procedure was performed twice. The longitudinal lengths of the deployed coil mass and volume embolization rates (VERs) at the embolization site obtained using the 3 techniques were compared statistically.

**Results:**

The longitudinal lengths of the deployed coil mass were 26 mm (range, 21–34 mm), 10 mm (8–14 mm), and 5 mm (5–5 mm) in proximal BT, distal BT, and DBT, respectively. The median VERs were 15.9% (12.2–19.4%), 41.4% (29.6–51.8%), and 82.9% (82.9–82.9%), respectively. Significant differences in the lengths and VERs were observed among the 3 techniques (*p* < 0.001).

**Conclusion:**

DBT achieved the tight packing of a hydrocoil in a short segment of an experimental vascular model compared with proximal BT and distal BT, suggesting DBT as the optimal embolization technique in this model.

## Introduction

Coil embolization of the parent artery in a short segment is usually difficult because of its fast arterial flow and the difficulty in preserving its branch flow. Recently, surgeons have performed embolization of the common hepatic artery for preoperative arterial flow alteration in distal pancreatectomy with en bloc celiac axis resection (i.e., DPCAR) for advanced pancreatic body cancer [1, 3, 6]. This procedure aims to prevent liver failure from the sudden stoppage of hepatic arterial flow by ligating the common hepatic artery. Coil embolization is occasionally difficult to perform using several techniques such as the scaffold technique, double-interlock technique, or balloon-assisted technique [1, 8]. In this context, we assumed that the use of 2 balloons could achieve a tightly packed short-segment coil embolization. We deduced that if a rigid coil could be coiled tightly, this technique may be potentially performed clinically. Incidentally, a hydrocoil is more rigid than other coils, which makes it difficult to use [2].

In this study, we evaluated the feasibility of achieving a tightly packed short-segment coil embolization in an experimental vascular model using the proximal (proximal BT), distal (distal BT), and double-balloon techniques (DBT). We placed a hydrocoil between 2 balloons for DBT and compared the coil embolization achieved with those of the distal and proximal BTs.

## Materials and Methods

The Ethics Committee of our institution approved the research protocol and waived the need for a permit being an experimental study.

### Vascular Model

**Fig. 1:**
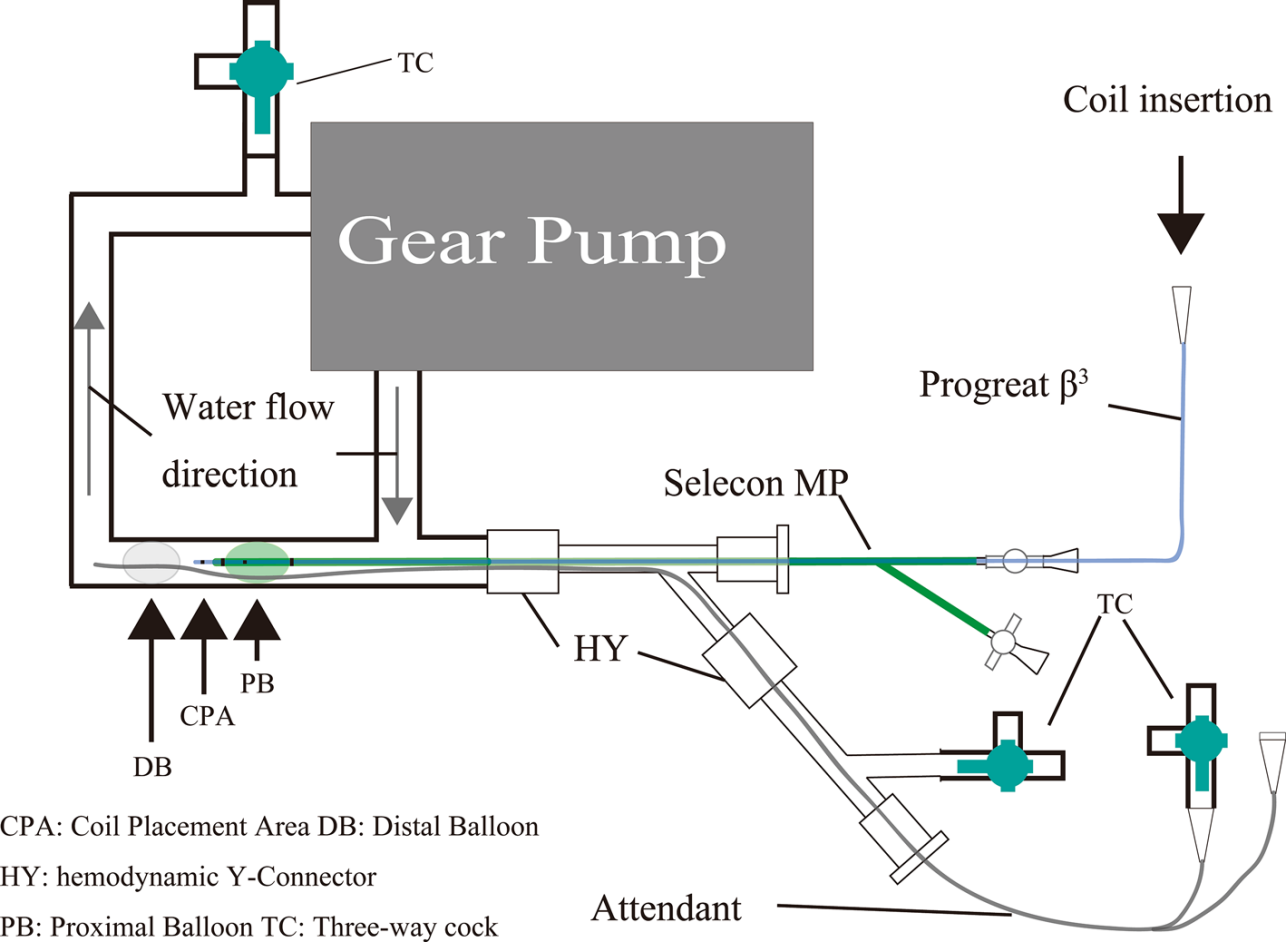
Schema of the experimental vascular model

We prepared a closed vascular model with a double hemostatic Y-connector as shown Fig. 1. The model was made up of a polyvinyl chloride tube of 6 mm internal diameter (Terumo, Tokyo, Japan) and a gear pump (TG-30S-PU; Tsukasa Denko, Tokyo, Japan). The model was perfused with a buffered solution prepared by dissolving 10 phosphate-buffered saline tablets (TakaraBio, Shiga, Japan) in 1 L of distilled water, and its flow rate was 3 ml/sec.

### Balloon Technique

**Fig. 2:**
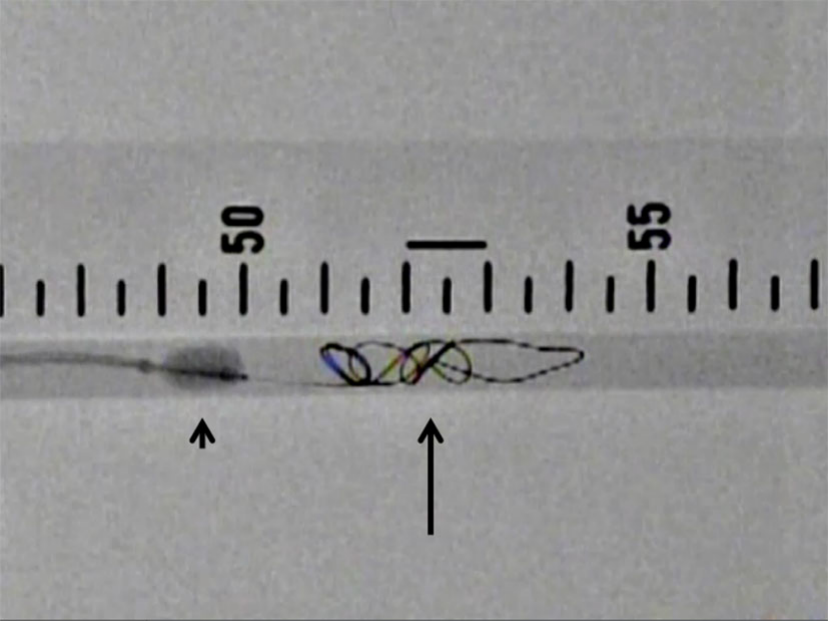
Proximal balloon technique (proximal BT): deployment of Azur (*arrow*) over an inflated Selecon MP balloon catheter (*arrow head*)

**Fig. 3:**
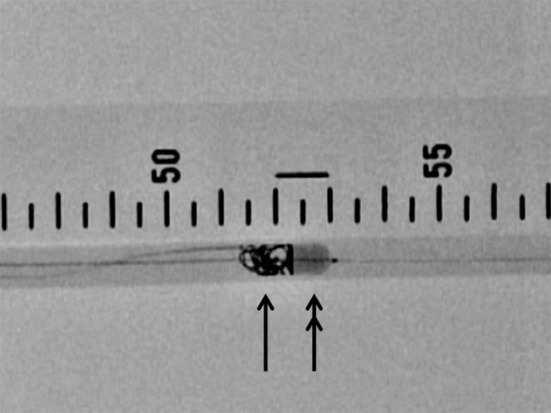
Distal balloon technique (distal BT): deployment of Azur (*arrow*) just before the Attendant (*double arrow*)

**Fig. 4:**
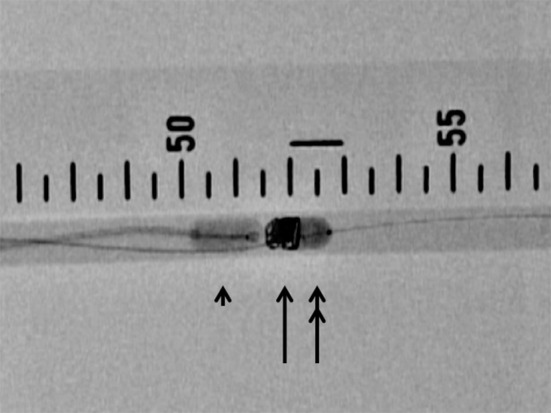
Double-balloon technique (DBT): deployment of Azur (*arrow*) between the Selecon MP balloon catheter (*arrow head*) and the Attendant (*double arrow*)

We performed 3 balloon techniques as follows: (1) proximal BT which involved proximal balloon inflation and coil deployment over the balloon (Fig. 2), (2) distal BT which involved distal balloon inflation and coil deployment at the proximal side of the inflated balloon (Fig. 3), and (3) DBT which involved the inflation of 2 balloons and coil deployment between the 2 balloons (Fig. 4).

The size of the proximal balloon catheter was 5.2 French (Selecon; Terumo), and the size of the distal balloon catheter was 3.0 French (Attendant; Terumo). These 2 catheters were inserted dependently from a branch of the vascular model using 2 Y-connector (Fig. 1). The maximum diameters of the proximal and distal balloons were 10 mm and 8 mm, respectively. In DBT, the 2 balloons of the balloon catheters were set at a distance of 5 mm. After inflating the balloons in each method, complete fluid flow stoppage was checked. The fluid flow stoppage showed no leakage from the opening of the distal three-way stopcock. Then, the gear pump was stopped. Also, the gear pump was stopped in distal BT and proximal BT after determining that there was no leakage. We used a 10-mm-diameter and 200-mm-long 2-dimensional helical-shape hydrocoil (Azur; Terumo) and deployed it through a microcatheter (Progreat *β* ^3^; Terumo). Azur was used as it achieves 5 times the volume after detachment according to a previous report [7], and it has a higher volume embolization rate (VER). The microcatheter was inserted through a 5.2 French Selecon balloon.

**Fig. 5:**
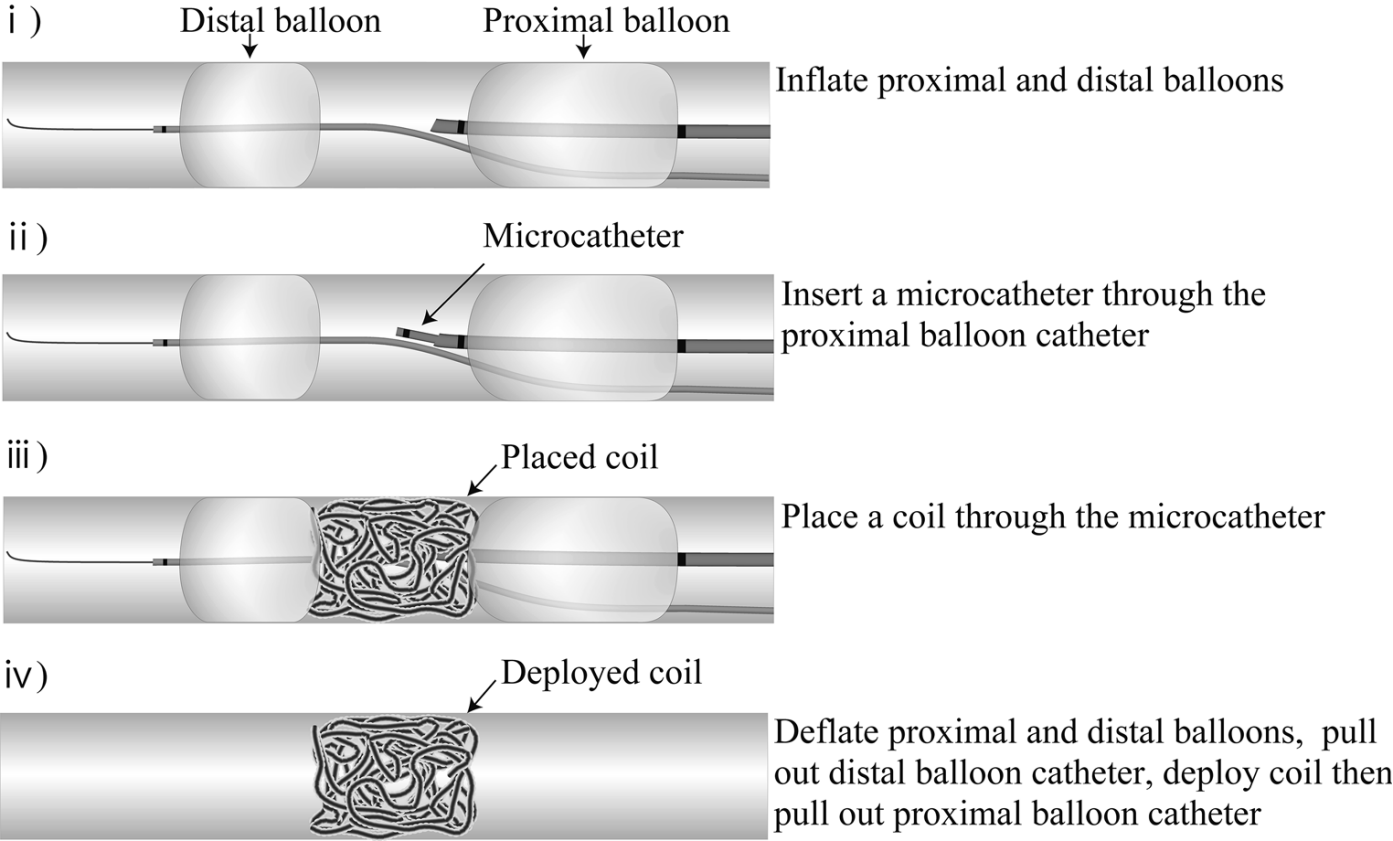
Schema of the double-balloon method

In distal and proximal BT, we deflated the balloon after deployment of a coil and pulled out balloon catheter. In DBT, we placed coil between 2 balloons (Fig. 5). We deflated proximal and distal balloons, pulled out distal balloon catheter, deployed a coil, and pulled out proximal balloon catheter. Then, we restarted the gear pump.

We observed whether coil migration occurred or not while placement of a coil to restarting the gear pump.

### Evaluation and Analysis

Five interventional radiologists with 25 years (2 doctors), 10, 3, and 1 year of experience participated in this study. They performed each procedure twice. The longitudinal length of the deployed coil mass was measured, and VER was calculated using the following equation:

$${\text{VER}} = {{\left\{ {{\text{volume of the embolized coil }}\left( {\text{VEC}} \right)} \right\}} \mathord{\left/ {\vphantom {{\left\{ {{\text{volume of the embolized coil }}\left( {\text{VEC}} \right)} \right\}} {\left\{ {{\text{volume of the parent artery }}\left( {\text{VPA}} \right)} \right\}}}} \right. \kern-0pt} {\left\{ {{\text{volume of the parent artery }}\left( {\text{VPA}} \right)} \right\}}} .$$

**Fig. 6:**
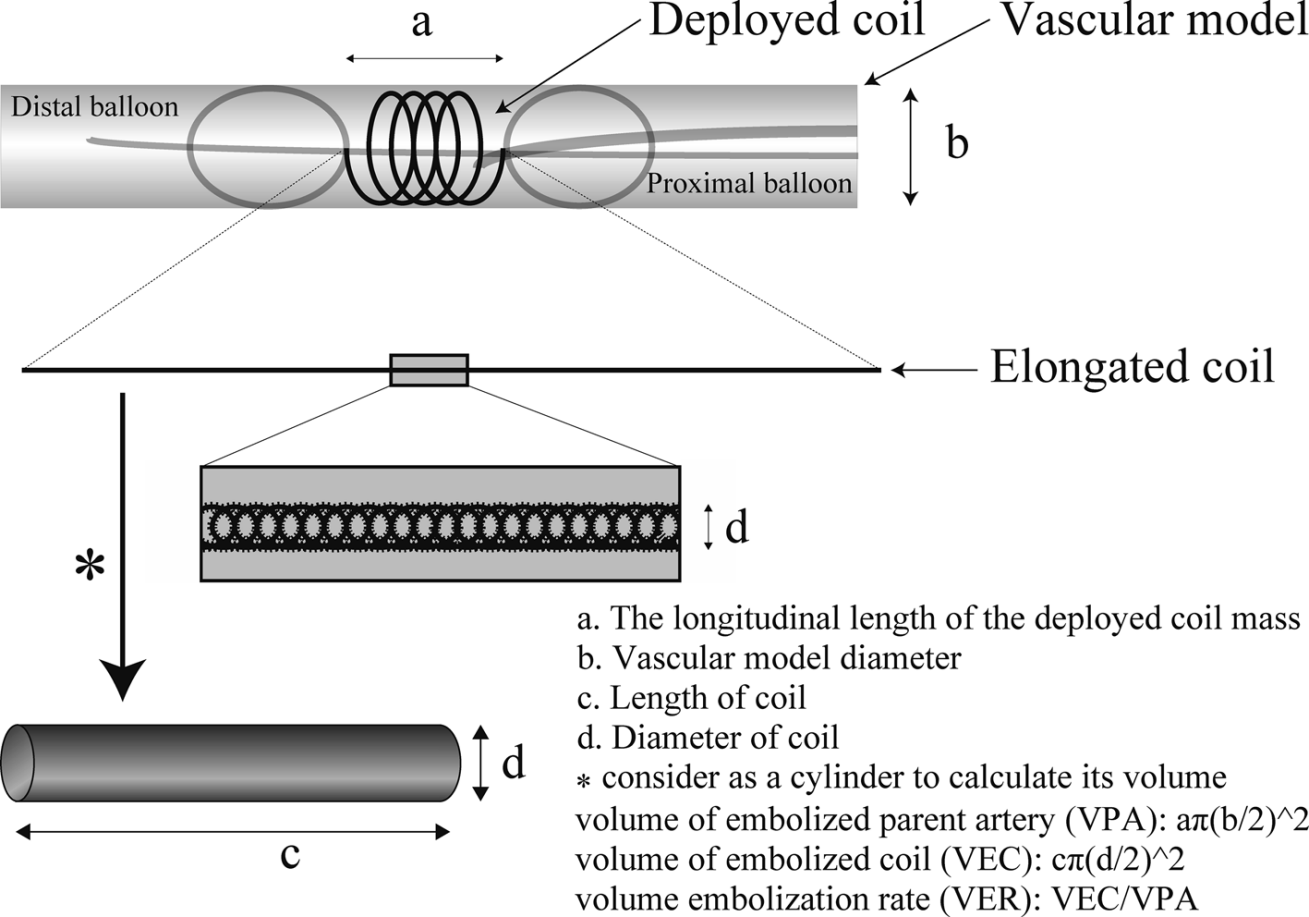
Schema of the explanation for the coil volume and formulas mentioned in the manuscript

VEC is calculated approximately on the supposition that the coil is a cylinder [9] (Fig. 6). VPA is considered as the volume of the vascular lumen where the coil mass was placed. VEC and VPA were calculated as follows:

$${\text{VEC}} = \pi \, \times \, \left( {{{\text{diameter of coil}} \mathord{\left/ {\vphantom {{\text{diameter of coil}} 2}} \right. \kern-0pt} 2}} \right)^{2} \times \, \left( {\text{length of coil}} \right) ,$$

$${\text{VPA}} = \pi \, \times \, \left( {{{6\,{\text{mm}}} \mathord{\left/ {\vphantom {{6\,{\text{mm}}} 2}} \right. \kern-0pt} 2}} \right)^{2} \times \, ({\text{longitudinal length of deployed coil mass}}) .$$

The bare coil volume of Azur (diameter, 10 mm; length, 10 cm) is 21.307 mm^3^, and the full-expanded volume is 117.15 mm^2^ after the hydrogel is fully expanded. Therefore, VER was calculated using the following formula:

$${\text{VER}} = {{117.15} \mathord{\left/ {\vphantom {{117.15} {\text{VPA}}}} \right. \kern-0pt} {\text{VPA}}} .$$

After 5 min which is considered to be the manipulating limit, we deflated the distal balloon and pulled the balloon. We continuously deflated the proximal balloon and restarted the gear pump. The longitudinal length of the deployed coil mass among the 3 balloon techniques was evaluated using the Kruskal–Wallis test. Correlations between experience and the longitudinal length of the deployed coil mass were analyzed using the Chi-square test. Each statistical value was considered to be significant when the *p* value was < 0.05.

## Results

In all the procedures, the balloons were placed and inflated in the intended area and the fluid flow was stopped completely.

The median and range longitudinal lengths of the deployed coil mass were 26 mm (11–34 mm), 9.5 mm (8–12 mm), and 5 mm (5–5 mm) in proximal BT, distal BT, and DBT, respectively (Table 1). A significant difference was observed between the DBT and the other techniques (*p* < 0.001). The median VERs were 15.9% (12.2–19.4%), 41.4% (29.6–51.8%), and 82.9% (82.9–82.9%), respectively. The VER of DBT was significantly higher than those of the other balloon techniques (*p* < 0.001). The longitudinal length of the deployed coil mass was not significantly related to experience.

**Table 1:** Longitudinal lengths of deployed coil mass with proximal, distal and double balloon technique by 5 various experienced interventional radiologists

Doctor	Experience (years)	Procedure	Proximal BT (mm)	Distal BT (mm)	DBT (mm)
1	25	1	34	11	5
		2	33	10	5
2	25	3	21	12	5
		4	32	9	5
3	10	5	21	9	5
		6	24	11	5
4	3	7	24	10	5
		8	25	8	5
5	1	9	32	9	5
		10	27	8	5

*Proximal BT* proximal balloon technique, *Distal BT* distal balloon technique, *DBT* double-balloon technique

Coil migration was not observed during procedures after placement of a coil even if we deflated the balloons and pulled the distal side balloon, or when we restarted the gear pump with fluoroscopy.

## Discussion

The scaffold technique and dual interlock technique are well-known coil embolization techniques [1]. However, although these techniques have been well studied, they have potential shortcomings such as (1) difficultly in determining the embolization length, and (2) there is a risk of coil migration as flow control is not achieved with a balloon. We also thought of making a space for putting the coil to prevent its migration. The present study supports the previous study of Takasaka et al. [8] which showed that distal BT was superior to proximal BT in terms of the length of the coil. Notably, our results demonstrated that DBT enabled the use of a shorter coil than distal BT.

In the present experimental study, DBT could achieve optimal embolization in terms of the tight packing of the hydrocoil in a short segment and the high VER. We used a hydrocoil with the intention of achieving a high VER and demonstrating rigidity [4] [2]. Although the rigidity was a disadvantage for tight packing in the short segment, DBT could achieve optimal embolization. We are also planning to apply DBT to other kinds of coils in our subsequent studies to clarify the feasibility of this technique with other coils.

Previous studies have reported that the optimal coil size was about 1.5–2 times the diameter of or 1–2 mm larger than the parent artery regardless of balloon assistance or not [2, 5, 8]. Therefore, we selected a 10-mm-diameter helical coil because the diameter of the parent artery model was 6 mm. Despite the stiffness of Azur, DBT was found to be the best technique among the 3 techniques in terms of the length of the coil regardless of the operator experience. We therefore consider that DBT is feasible for operators with an average experience.

The dual microcatheter-dual interlocking detachable coil technique or distal balloon technique was reported as means of parent artery occlusion [1, 8]. Although these techniques are intended to protect coil migration, controlling the length and detailed place of the embolized coil is relatively difficult. We believe that DBT is an appropriate indication for the short-segment embolization of a parent artery compared with the above-mentioned techniques because operators can control the length of the embolized coil at will.

The present study has several limitations. *First*, the experimental environment differed from the natural environment of the human body; hence, it is still unclear whether DBT can achieve the same results when applied to humans. *Second*, although we used 10-mm-diameter Azur for a 6-mm-diameter experimental vascular model according to a previous report, the appropriate coil diameter for DBT needs to be clarified. *Third*, we stopped the gear pump after inflating the balloons because water leakage occurred inside the gear pump. Thus, our data analysis might not be accurate because of the lack of fluid pressure and suspension derived from the gear pump and collateral pathways. These elements might affect coil deployment manipulation and coil mass formation. To verify the problems mentioned above, we have to evaluate them in the animal experiments and human clinical trials.

In conclusion, DBT achieved the tight packing of a hydrocoil in a short segment of an experimental vascular model compared with proximal BT and distal BT, suggesting DBT as the optimal embolization technique in this model.
