# Erratum to: Short-Segment Coil Embolization Using a Double-Balloon Technique in an Experimental Vascular Model

**DOI:** 10.1007/s00270-017-1647-8

**Published:** 2017-05-25

**Authors:** Daisuke Yunaiyama, Toru Saguchi, Tomohisa Moriya, Natsuhiko Shirota, Jun Otaka, Koichi Tokuuye, Yuichi Nagakawa, Akihiko Tsuchida, Kazuhiro Saito

**Affiliations:** 1 Department of Radiology, Tokyo Medical University, 6-7-1 Nishishinjuku, Shinjuku-ku, Tokyo, Japan; 2 Department of Gastrointestinal and Pediatric Surgery, Tokyo Medical University, Tokyo, Japan

## Erratum to: Cardiovasc Intervent Radiol DOI 10.1007/s00270-017-1589-1

The article was originally published electronically on the publisher’s internet portal (currently SpringerLink) on 08 February 2017 without open access.

With the author(s)’ decision to opt for Open Choice, the copyright of the article changed on 23 May 2017 to © The Author(s) 2017 and the article is forthwith distributed under the terms of the Creative Commons Attribution 4.0 International License. The original article was corrected.

